# A comprehensive update of genotype–phenotype correlations in PMM2-CDG: insights from molecular and structural analyses

**DOI:** 10.1186/s13023-025-03669-5

**Published:** 2025-04-30

**Authors:** Tiago Oliveira, Ricardo Ferraz, Luísa Azevedo, Dulce Quelhas, João Carneiro, Jaak Jaeken, Sérgio F. Sousa

**Affiliations:** 1 LAQV/REQUIMTE, BioSIM, Department of Biomedicine, Faculty of Medicine, University of Porto, Alameda Prof. Hernâni Monteiro, Porto, Portugal; 2 Centre for Translational Health and Medical Biotechnology Research (TBIO), Chemical and Biomolecular Sciences, School of Health, Polytechnic Institute of Porto, Porto, Portugal; 3 LAQV/REQUIMTE, Department of Chemistry and Biochemistry, Faculty of Sciences, University of Porto, Campo Alegre, Porto, Portugal; 4 Chemical and Biomolecular Sciences, School of Health, Polytechnic Institute of Porto, 4200072 Porto, Portugal; 5 ITR - Laboratory for Integrative and Translational Research in Population Health, Porto, Portugal; 6 Unit for Multidisciplinary Research in Biomedicine, ICBAS, UP, Porto, Portugal; 7 Unidade de Bioquímica Genética, Serviço de Genética Laboratorial, Centro de Genética Médica, Clínica de Genética e Patologia, Unidade Local de Saúde de Santo António, Porto, Portugal; 8 Centro Referência Doenças Hereditárias do Metabolismo, Unidade Local de Saúde de Santo António, Porto, Portugal; 9 CIIMAR, Interdisciplinary Centre of Marine and Environmental Research, Terminal de Cruzeiros Do Porto de Leixões, University of Porto, Av. General Norton de Matos, Matosinhos, Portugal; 10 Center for Metabolic Diseases, University Hospital Gasthuisberg, KU Leuven, Leuven, Belgium

**Keywords:** PMM2, PMM2-CDG, Genotype–phenotype correlations, Molecular analysis, Missense variants

## Abstract

PMM2-CDG (phosphomannomutase 2-deficiency) is the most prevalent N-glycosylation disorder and results from impairments of PMM2 activity. This disease presents a large variety of pathogenic variants, which cause a wide phenotypical spectrum. This diversity, together with the low number of affected patients, raises the challenge of determining genotype–phenotype correlations in PMM2-CDG. This type of correlation could be highly significant in determining disease progression, prognosis, severity and in developing genome-personalized therapies. Structural analyses offer a valuable approach for assessing the pathogenic mechanisms within the PMM2 protein structure at a molecular level. Such an approach can reveal novel insights into the consequences of missense variants and their relationship with patients'phenotype. In this comprehensive review, we evaluate at a structural level 41 missense mutations in PMM2-CDG, examining their phenotypical characteristics and clinical severity, protein properties and interference at the enzymatic level. This work broadens the understanding of the intricate relationships between genotype and clinical manifestations of PMM2-CDG.

## Introduction

Congenital disorders of glycosylation (CDG) are a large group of genetic diseases due to variants in proteins involved in glycosylation [54, 57].

PMM2 (phosphomannomutase 2; MIM# 601785) catalyses the interconversion of glucose- 6-phosphate (G6P) to glucose- 1-phosphate (G1P) and, majorly, mannose- 6-phosphate (M6P) to mannose- 1-phosphate (M1P), requiring glucose- 1,6-bisphosphate (G16BP) or mannose- 1,6-bisphosphate (M16BP) as activators [48]. M1P is a precursor of guanosine diphosphate mannose (GDPM), which provides mannose for the synthesis of dolichol-phosphate-mannose (Dol-P-M), a lipid-linked oligosaccharide (LLO) precursor of the N-glycosylation [3]. Briefly, this metabolic pathway involves the covalent attachment of a glycan precursor (Dol-P-M) to a protein, more precisely to the nitrogen atom of the amido group side chain of a proteins’ asparagine residues at an Asn-X-Ser/Thr site (X represents any residue except proline) [16, 62].

*PMM2* gene variants result in PMM2-CDG (phosphomannomutase 2—deficiency; MIM# 212065), the most prevalent N-glycosylation disorder, first reported by Jaeken et al. [34]. It is an autosomal recessive disease and a CDG-I, with a wide spectrum of clinical manifestations [43] related to defects in the biosynthesis and transfer of LLOs [75]. This process results in hypoglycosylation of numerous glycoproteins, leading to multi-organ involvement. The primary impact is on the nervous system [3] causing ataxia, epilepsy, hypotonia, microcephaly, peripheral neuropathy, stroke-like episodes and developmental disability [44]. Other common symptoms of PMM2-CDG are abnormal fat distribution, facial dysmorphism, inverted nipples, strabismus, coagulation and endocrine anomalies [26]. The phenotype severity ranges from mild to a severe neonatal phenotype with multi-organ involvement [3].

More than 1000 patients have been reported with an estimated birth incidence between 0.06/100,000 and 5/100,000 [54]. Some 20% of patients die in the first four years of life [80]. Unfortunately, only symptomatic treatment is available [29, 44, 47].

A rather high number of disease-causing variants has been reported [19, 56, 59, 66, 71]. The combination of a limited number of reported patients and the need for genotype–phenotype correlations can provide insights into disease aetiology, prognosis, progression, and severity [27], as well as allow the development of genome-personalized therapies [61]. According to Quelhas et al. [56], structural analyses can be a tool to discover novel insights about the molecular impact of missense mutations in PMM2-CDG. Expanding upon this possibility, this review correlates the molecular basis of the impact of 41 missense mutations on PMM2 structure and/or function combined with data from phenotypical outcomes, clinical severity and enzymatic activity.

## PMM2 missense variants

To date, 142 PMM2 variants have been identified, 133 of which are missense, being p.Arg141His (c.422G > A) the most frequent [43]. Despite the high number of carriers, no homozygous (p.Arg141His/p.Arg141His) patients have been described, suggesting that it would result in the absence of protein activity, which is incompatible with life [66]. In fact, the majority of PMM2-CDG patients are compound heterozygotes with one hypomorphic variant in one allele and one loss-of-function variant in the other allele [71]. A novel research by Briso-Montiano et al. [12], predicted the impact of missense variants on the PMM2 experimental structure and classified them according to the potential mechanism of impact on PMM2 function as follows: (1) activator binding, (2) catalysis, (3) dimerization, (4) folding of the cap and (5) core domains, and (6) linker- 2 flexibility, which could affect the movement of the core domain in the catalytical process.

**Fig. 1:**
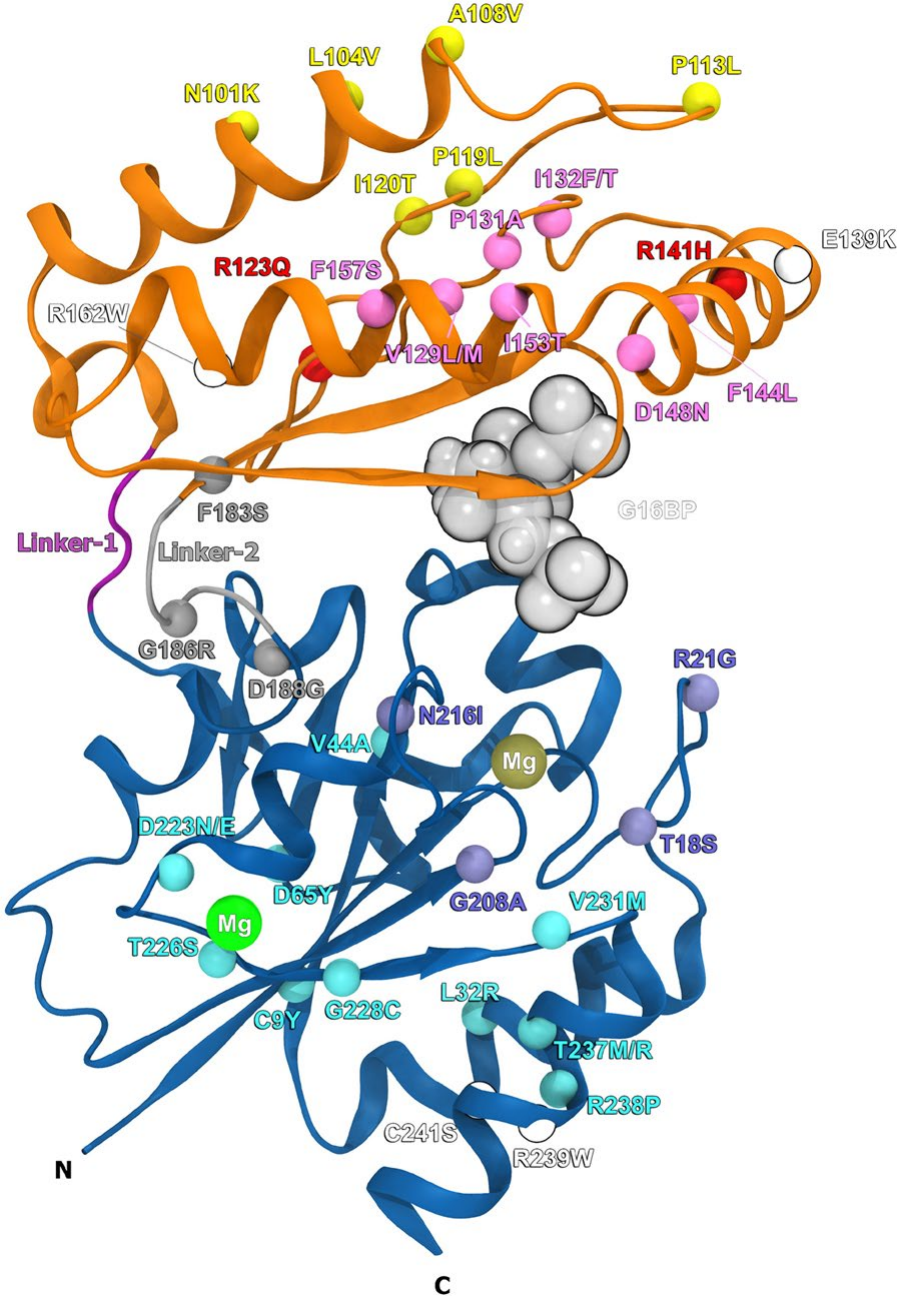
Mapping of missense PMM2 variants. Only one subunit (A) of PMM2 structure is represented. The core and cap domains are coloured blue and orange, respectively. The catalytical Mg2+ and the structural Mg2+ are coloured, respectively, tan and green. The activator (G16BP) is represented in Van der waals, in a semi-diffuse grey representation. Linker- 1 and linker- 2 are coloured, respectively, magenta and silver. The positions of mutated residues in PMM2-CDG are represented as beads and coloured by their potential mechanism of impact on protein function [12]. Accordingly, variants that are predicted to impact the folding of the cap and core domains are coloured mauve and cyan, respectively. Variants that could affect the linker- 2 are represented in grey, and variants that could disrupt the binding of the activator to the protein are coloured red. Variants that could disrupt the dimerization are coloured yellow. Finally, variants with an uncertain predicted mechanism of impact in PMM2 function are coloured white

In the following sections, we present a concise overview of the phenotypes observed in PMM2-CDG patients, the associated disease-causing variants and the effects at the structural or functional levels. These analyses were based on the available X-ray structures of PMM2 [12] (particularly structure 7O4G PDB for the wild-type protein), upon in-depth inspection with PyMOL and its wizard mutagenesis plug-in. The studies that analysed the structure [12, 66] were used as a basis of comparison. The missense PMM2 variants discussed herein were selected based on their classification as pathogenic/likely pathogenic in the ClinVar database, a public archive of reports of the relationships among human variations and phenotypes [41]. All variants reviewed in this work are listed in Table 1, and they are represented in the PMM2 7O4G PDB monomer structure in Fig. 1. A summary of all phenotypes with respect to each genotype, including number of patients per genotype is stated in Table 2.

**Table 1:** Forty-one disease-associated variants of PMM2-CDG

Variant	Structure		
hPMM2	cDNA	Classification	Location
p.Cys9 Tyr	c.26G > A	Folding	Core domain
p.Thr18Ser	c.53 C > G	Catalysis	
p.Arg21Gly	c.61G > C	Catalysis	
p.Leu32 Arg	c.95 TA > GC	Catalysis	
p.Val44 Ala	c.131 T > C	Folding	
p.Asp65 Tyr	c.193G > T	Folding	
p.Asn101Lys	c.303 C > G	Dimerization	Cap domain
p.Leu104 Val	c.310 C > G	Dimerization	
p.Ala108 Val	c.323 C > T	Dimerization	
p.Pro113Leu	c.338 C > T	Dimerization	
p.Phe119Leu	c.355 C > A	Dimerization	
p.Ile120 Thr	c.359 C > A	Dimerization	
p.Arg123Gln	c.368 A > C	Activator binding	
p.Val129Leu	c.385G > A	Folding	
p.Val129Met	c.385G > A	Folding	
p.Pro131 Ala	c.391 C > G	Folding	
p.Ile132Phe	c.394 A > T	Folding	
p.Ile132 Thr	c.395 T > C	Folding	
p.Glu139Lys	c.415G > A	Uncertain	
p.Arg141His	c.422G > A	Activator binding	
p.Phe144Leu	c.430 T > C	Folding	
p.Asp148 Asn	c.442G > A	Folding	
p.Ile153 Thr	c.458 T > C	Folding	
p.Phe157Ser	c.470 T > C	Folding	
p.Arg162 Trp	c.484 T > C	Uncertain	
p.Phe183Ser	c.548 T > C	Linker- 2	
p.Gly186 Arg	c.556G > A	Linker- 2	
p.Asp188Gly	c.563 A > G	Linker- 2	
p.Phe207Ser	c.620 T > C	Folding	Core domain
p.Gly208 Ala	c.623G > C	Catalysis	
p.Asn216Ile	c.647 A > T	Catalysis	
p.Asp223 Asn	c.667G > A	Folding	
p.Asp223Glu	c.669 C > G	Folding	
p.Thr226Ser	c.677 C > G	Folding	
p.Gly228 Cys	c.682G > T	Folding	
p.Val231Met	c.691G > A	Folding	
p.Thr237 Arg	c.710 C > G	Folding	
p.Thr237Met	c.710 C > T	Folding	
p.Arg238Pro	c.713G > C	Folding	
p.Arg239 Trp	c.715 A > T	Uncertain	
p.Cys241Ser	c.722G > C	Uncertain	

All 41 missense variants are classified according to their predicted mechanism of impact on PMM2 function, and their location in the protein structure is referred

**Table 2:** Genotype–Phenotype Correlations in PMM2-CDG

Genotype		Phenotype		No.Patients	References
Mutation	Combination	Severity	Characteristics		
C9Y	D148 N	Mild	Ataxia, Failure to Thrive (FTT), Gastroesophageal (GE) reflux, Hydronephrosis, Hypotonia, Impaired development, Lipodystrophy, Microcephaly, Mild vermal cerebellar atrophy	1	[79]
T18S	V67G	Mild	Ataxia, Impaired development, Intention tremors, Mild dysmorphic features, Normal neuroimaging	1	[21]
R21G	R21G	Severe	Severe lymphatic oedema	1	[74]
L32R	R123Q	Mild	Extra-neurological features, Mild intellectual disability, Moderate cerebellar hypoplasia, Visual defects	1	[7]
	R141H	Mild	Ataxia, Cerebellar hypoplasia, Dysmorphic features, Early esotropia, Hypotonia, Impaired development, Microcephaly, Mild intellectual disability, Osteoporosis or osteopenia, Small size according to weight, Titubation, Visual defects	10	[7, 22, 79]
	F157S	Mild	Mild intellectual disability, Moderate to severe cerebellar hypoplasia, Visual defects	2	[7]
	T237M	Mild	Extra-neurological features, Microcephaly, Moderate intellectual disability, Visual defects	2	[7]
V44 A	D65Y	Mild	Ataxia, Cerebellar hypoplasia, Growth failure, Mental retardation	1	[73]
	R123Q	Severe	Convulsions, Incapacity of locomotion, Intellectual disability, Multisystem involvement, Peripheral neuropathy, Pigmentary retinopathy, Recurrent infections, Spine involvement	1	[31]
	R141H	Variable	Aortic thrombosis – early death (severe) or Similar to V44 A/R123Q (mild)	2	[31, 73]
	F207S	Mild	Brain atrophy, Hepatopathy, Hypotonia	1	[73]
D65Y	D65Y	Moderate	N/A	1	[60]
	R123Q	Severe	Early death	2	[60]
	R141H	Severe	Early death	4	[23, 60]
	F157S	Severe	Early death	2	[11, 64]
N101 K	324 del g	N/A	N/A	1	[46]
L104 V	D12H	Mild	FTT, Hypergonadrotropic hypogonadism, Hypertelorism, Hypotonia, Kyphoscoliosis, Mild hyperprolactinemia and hyperthyroglobulinemia, Primary ovarian insufficiency, Strabismus	1	[45]
	IVS1–1G > A	Severe	Anaemia, Coagulopathy, GE reflux, Hypertrophic cardiomyopathy, Hypoalbuminemia, Hypoglycaemia, Pericardial effusion	1	[78]
A108 V	c.511 dupA	Mild	Moderate cerebellar hypoplasia	1	[7]
	70 kb-deletion	Mild	Cerebellar vermis hypoplasia, Esotropia, Feeding problems, Hyperlaxity, Hypotonia, Inverted nipples, No facial dysmorphism	1	[7]
	R123Q	Severe	Convulsions, FTT, Inability to walk, Multiorgan involvement, Peripheral neuropathy, Recurrent infections, Retinitis pigmentosa, Scoliosis, Severe delayed development	1	[31]
P113L	R141H	Severe	Ataxia, Blood coagulation abnormalities, Brain hypoplasia, Disturbed development, Disturbed psychomotor abilities, Hyporeflexia, Low levels of thyroid hormones, Retinitis pigmentosa, Retracted nipples, Severe hyperinsulinemic hypoglycaemia, Strabismus	5	[10, 13, 23 ]
	F157S	Moderate	Cerebellar hypoplasia, Hypotonia, Mild delayed development, Strabismus	1	[31]
	T237M	Severe	Convulsions, FTT, Inability to walk, Multiorgan involvement, Peripheral neuropathy, Recurrent infections, Retinitis pigmentosa, Scoliosis, Severe delayed development	2	[31]
F119L	R141H	Severe	Early dead (6 patients), Feeding difficulties, FTT, Impaired development, Inverted nipples, Liver dysfunction, Pericardial effusion, Postnatal growth failure, Progressive brain atrophy, Serious hypotonia, Subcutaneous fat pads, Unable to walk without support	51	[23, 36, 37]
I120 T	G228 C	Mild	Ataxia, Blood coagulation abnormalities, Cerebellar vermis hypoplasia, Disturbed motor development, FTT, Impaired speech, Strabismus	1	[72]
	V231M	Mild	Blood coagulation abnormalities, Hemiparesis, Seizures	1	[24]
R123Q	C241S	Mild	Ataxia, Cerebellar vermis hypoplasia, Esotropia, GE reflux, Hypotonia, Impaired development and psychomotor abilities, Mild brain atrophy, Mild lipodystrophy, Retracted nipples, Tremors	2	[14, 79]
V129L	161_162 insG	Severe	Cardiological failure, Early death, Hydrops, Hydrops fetalis and oedema, during foetal development, Hypertrophic cardiomyopathy, Hypoglycaemia, Severe amegakaryocytic thrombocytopenia	2	[81]
V129M	L32R	Mild	Moderate cerebellar hypoplasia, Moderate intellectual disability, Visual defects	2	[7]
	R141H	Severe	Abnormal levels of blood coagulation factors, Ataxia, Brain atrophy, Hepatic steatosis, Microcephaly, Osteopenia, Severe cerebellar hypoplasia, Stroke-like episodes are probable, Visual defects	7	[7, 8, 23]
	IVS3 + 2 C > T	Severe	Early death	1	[53]
P131 A	R141H	N/A	N/A	1	[42]
I132 F	E197 A/I132 F	N/A	N/A	1	[42]
I132 T	R123Q	Mild	Areflexia, Brain hypoplasia, Disturbed psychomotor abilities, Dysmorphism, FTT, Hepatomegaly, Hypotonia, Pericardial inflammation, Strabismus, Tubulopathy	1	[23]
	R141H	Mild	Areflexia, Brain hypoplasia, Facial dysmorphism, Fat pads, Feeding problems, FTT, Hepatomegaly, Hyperprolactinemia, Hypotonia, Recurrent seizures, Retarded development and psychomotor abilities, Strabismus, Tubulopathy	4	[7, 23, 30]
	F207S	Mild	GE reflux, Hepatomegaly, Hypothyroidism, Hypotonia, No fat pads, Normal neuroimaging	1	[67]
E139 K	R141H	Mild	Areflexia, Cerebellar atrophy, Disabled psychomotor capacities, Feeding problems, Hypoplasia, Hypotonia, Inverted nipples, Seizures	7	[23, 65, 76]
R141H	V43M	Mild	Disabled development, Esotropia, Hypermetropia, Hypotonia, Increased serum transaminases, Mild dysmorphism	1	[9]
	A108 V	Mild	Areflexia, Cerebellar hypoplasia, Diarrhoea, Hypotonia, Psychomotor, retardation, Strabismus	1	[23]
	G228 C	Mild	Brain haemorrhage, Malignant brain tumour, Typical PMM2-CDG phenotype	1	[68]
F144L	R238P	Mild	Ataxia, Dysmorphic facial traits, Feeding difficulties, Hypergonadotropic hypogonadism, Impaired motor development, Low height, Mild brain atrophy, Muscular hypotonia, Unable to speak, Rapid onset after birth, Recurrent stroke-like episodes, Seizures and hemiparesis due to several head injuries, Severe delayed development, Strabismus, Tremors, Only walk with support	2	[40]
	Y229S	Mild	Ability to walk unaided, Ataxia, Brain atrophy, Disabled mental development and psychomotor abilities, Hypotonia, Increased serum transaminases, Recurrent stroke-like episodes	2	[40]
D148 N	F183S	Mild	Disabled mental development, Dysmorphism, Fat pads, Feeding difficulties, Hypotonia, Spine abnormalities, Strabismus	1	[33]
I153 T	R123Q	Severe	Convulsions, FTT, Inability to walk, Multiorgan involvement, Peripheral neuropathy, Recurrent infections, Retinitis pigmentosa, Scoliosis, Severe delayed development	1	[31]
	R141H	Severe	Brain hypoplasia, Diarrhoea, Disturbed psychomotor abilities, Hypotonia, Pericarditis, Renal cysts, Seizures, Strabismus	1	[23]
F157S	V231M	Severe	Early death	1	[60]
	C241S	Mild	Cerebellar hypoplasia, Hypotonia, Mild delayed development, Strabismus	2	[31, 60]
R162 W	R123X	Moderate	N/A	1	[60]
	R141H	Moderate	Cerebellar hypoplasia, Hypotonia, Mild delayed development, Strabismus	1	[31]
F183S	R141H	Mild	Mild liver dysfunction, Mild peripheral neuropathy, Neurological involvement	7	[25]
	F183S	Mild	Mild liver dysfunction, Mild peripheral neuropathy, Neurological involvement	1	[25]
G186R	G186R	Mild	Most of common traits of PMM2-CDG were not apparent	2	[52]
D188G	L35X	Severe	Ascites, Early death, Fat pads, FTT, Hepatic involvement, Hypoalbuminemia, Inverted nipples, Pericardial effusion, Renal involvement, Strabismus, Thrombocytopenia	1	[70]
	R141H	Severe	Early death	4	[55]
F207S	Y67 C	Severe	Cerebellar hypoplasia, Dysmorphism, Early death, Hepatopathy in the neonatal period, Severe encephalopathy	1	[11]
	N216I	Mild	Cerebellar hypoplasia, Convulsions, Disabled psychomotor development, Obesity, Retracted nipples	1	[11]
G208 A	R141H	Severe	Anaemia, Coagulation abnormalities, Ventricular septum defect, Pericardial effusion, Hepatic and renal involvement, Seizures, Multiorgan failure—early death; Severe pericardial effusion, left ventricle failure—early death	2	[2, 32, 33]
N216I	R141H	Severe	Cerebellar hypoplasia, Extra-neurological features, Microcephaly, Moderate to severe intellectual disability, Stroke-like episodes, Visual defects	3	[7]
	N216I	Mild	Brain hypoplasia and dilatation of the 4 th ventricle, Hypotonia, Macrosomia, Mild psychomotor disability, Recurrent respiratory tract infections, Strabismus	1	[49]
D223E	N/A	N/A	Nearly total loss of PMM activity in fibroblasts	1	[38]
D223 N	G15E	Severe	Brain hypoplasia, Inability to walk without support, Intellectual disability, Severe microcephaly, Stroke-like episodes, Visual defects	1	[7]
	R141H	Severe	Microcephaly, Severely impaired intellectual development	1	[7]
T226S	R141H	Mild/moderate	Cerebellar hypoplasia, Hypotonia, Psychomotor disability, Strabismus	2	[23, 31]
G228 C	I120 T	Mild	Ataxia, Cerebellar hypoplasia with vermis hypoplasia, Disturbed fine motor skills, Dysarthric speech, Hypotonia, Strabismus	1	[72]
V231M	R141H	Severe	Incapability of walking without support, Microcephaly, Multisystem involvement (neurological manifestations, vomiting, and renal involvement), Severe cerebellar hypoplasia, Severe intellectual disability, Two early deaths derived from cardiological involvement, Two early deaths due to multiorgan involvement	8	[6, 7, 23]
	T237R	Severe	Cardiological and respiratory failure led to early death	1	[5]
	R239 W	Mild/moderate	Appropriate cognitive development	3	[72]
T237R	T237M/(T237R + A233 T)	Severe	Convulsions, FTT, Inability to walk, Multiorgan involvement, Peripheral neuropathy, Recurrent infections, Retinitis pigmentosa, Scoliosis, Severe delayed development	1	[31]
	C241S	Mild	Mild intellectual impairment, Moderate cerebellar hypoplasia	1	[7]
T237M	R141H	Mild	Ataxia, FTT, Impaired development, Mild dysmorphism, Mild retinal dysfunction, Severe muscle hypotonia, Tremors	1	[33]
	^a^	Mild	Abnormal fat distribution, Amblyopia, Dysmorphic traits, FTT, GE reflux, Impaired development, Inability to walk without support, Mild brain hypoplasia, Muscle hypotonia, Retracted nipples, Strabismus	1	[69]
R238P	R194X	Moderate	FTT, Recurrent non-epileptic seizures, Impaired development, Hepatomegaly, Increased serum transaminases, decreased serum ceruloplasmin	1	[17]
R239 W	V231M	Mild	Appropriate to mild delayed cognitive development, Cerebellar hypoplasia, Hypotonia, Strabismus	5	[31, 72]
C241S	R141H	Mild	Mild impaired motor development, No coagulation abnormalities, No hepatic dysfunction	7	[7, 23, 60, 72]

^a^c.565–571 delAGAGAT insGTGGATTTCC

### Cys9 Tyr (C9Y)

In the novel PMM2 structure (PDB code 7O4G), the Cys9 residue lies in sheet β1 in the core domain, and its replacement by a tyrosine would alter the proper folding of the core domain [12]. This missense variant reduces more than half of PMM2 activity and results in a more thermolabile protein [77]. Additionally, it was reported to cause a mild phenotype in a compound heterozygous (p.Cys9 Tyr/p.Asp148 Asn) male patient. The affected individual demonstrated ataxia, failure to thrive (FTT), hypotonia, microcephaly, impaired development, gastroesophageal (GE) reflux, hydronephrosis, lipodystrophy and mild vermal cerebellar atrophy [79].

### Thr18Ser (T18S)

The amino acid residue Thr18, located in the β1‐α1 loop (aa 12–24) in the core domain, establishes a hydrogen bond with Gly15 in the same region that coordinates the catalytical Mg^2+^. The replacement of a glycine by a serine would probably preserve the hydrogen bond but could result in the loss of some hydrophobic interactions [66]. Accordingly, Briso-Montiano et al. [12] predicted that this variant would displace the positioning of Asp12 and Asp14 residues that coordinate the Mg^2+^. This could destabilize the conformation of the β1‐α1 loop, affecting the catalysis. However, in vitro assays revealed that the p.Thr18Ser has a similar protein activity when compared to the wild-type PMM2 [42, 66]. Regarding clinical severity, a mild phenotype was reported in a compound heterozygous [p.Thr18Ser/p.Val67Gly (c.200 T > G)] six-year-old female patient with Australian ancestry. After birth, this patient presented mild dysmorphic features and impaired development. After six months of life, ataxia and intention tremors were apparent. However, neuroimaging remained normal [21].

### Arg21Gly (R21G)

According to a recent research by Briso-Montiano et al. [12], the Arg21 residue is situated in the core domain in the PMM2 structure. The missense p.Arg21Gly replacement could affect catalysis, since it could change the conformation of the β1‐α1 loop, resulting in improper positioning of Asp12 and Asp14 that coordinate the catalytic Mg^2+^. This variant was described in a homozygous (p.Arg21Gly/p.Arg21Gly) patient with severe lymphatic oedema [74].

### Leu32 Arg (L32R)

According to Briso-Montiano et al. [12], the hydrophobic Leu32 residue is located in helix α1 in the core domain of the PMM2 structure. The replacement for an arginine residue at amino acid residue 32 can lead to impaired folding of the core domain. The p.Leu32 Arg variant exhibits less than half of the enzymatic activity and has increased thermolability when compared to the WT protein [77]. This variant is prevalent in Italy and is typically associated with a mild neurological phenotype [7]. In the same study, Barone et al. [7] identified 12 compound heterozygous patients with this variant. Additionally, Coman et al. [22] reported three compound heterozygous (p.Leu32 Arg/p.Arg141His) patients, also exhibiting a mild phenotype. Finally, Westphal et al. [79] reported two patients with this variant, but one carried it heterozygosity with a single base pair deletion. Both presented early esotropia and impaired development. They also showed ataxia, titubation, and cerebellar hypoplasia. The male patient also showed congenital hypotonia and was small for his weight.

### Val44 Ala (V44 A)

Val44 residue resides in the c-terminus of sheet β2 in the core domain. Its substitution for a more permissive alanine could disrupt some hydrophobic interactions, causing unproper folding of the core domain in the PMM2 structure [12]. Moreover, Yuste-Checa et al. [82] reported that the p.Val44 Ala variant decreases protein activity despite showing a similar percentage of dimerization when compared to WT PMM2 protein. This missense variant, which probably contributes to a mild phenotype, was identified in two patients in compound heterozygosity with the p.Arg141His and p.Arg123Gln variants. In both cases, the patients showed a severe phenotype with typical clinical manifestations of PMM2-CDG, such as multisystem involvement, intellectual disability, incapacity of locomotion, pigmentary retinopathy, recurrent infections, spine involvement, peripheral neuropathy, and convulsions [31]. In addition, Vega et al. [73] also identified a compound heterozygous patient in conjunction with p.Arg141His who developed an aortic thrombosis, leading to an early fatal outcome. The same study also reported a compound heterozygous (p.Phe207Ser/p.Val44 Ala) patient, with brain atrophy, hypotonia and hepatopathy. Additionally, the authors also documented a compound heterozygous (p.Val44 Ala/p.Asp65 Tyr) patient that manifested ataxia cerebellar hypoplasia, mental retardation and growth failure.

### Asp65 Tyr (D65Y)

In the PMM2 structure, the Asp65 residue is located in the core domain. Its replacement by a tyrosine could disrupt some polar/charged interactions that maintain structure stability, leading to improper folding of this region [12]. The Asp65 Tyr variant is mostly restricted to Portugal and Spain, and it was reported in a homozygous (p.Asp65 Tyr/p.Asp65 Tyr) patient with a moderate phenotype [60]. Contrastingly, a severe phenotype characterized by early death in the first year of life was described in four patients, who were compound heterozygotes (two carriers of p.Asp65 Tyr/p.Arg123Gln, and the other two carrying p.Asp65 Tyr/p.Arg141His) [60]. A severe phenotype was also reported in a compound heterozygous (p.Asp65 Tyr/p.Phe157Ser) with multisystem failure and early fatal outcome [64]. This patient presented typical symptoms of PMM2-CDG, such as axial hypotonia, lipodystrophy, facial dysmorphism, arachnodactyly, and non-palpable gonads. The severity of this phenotype, with a particularly serious neonatal onset, was probably related to the p.Phe157Ser missense variant [64]. In accordance, Briones et al. [11] also reported a severe phenotype in a Spanish patient with the p.Phe157Ser/p.Asp65 Tyr genotype, which led to an early death possibly due to the lack of PMM2 residual activity. This patient showed a very typical PMM2-CDG phenotype.

### Asn101Lys (N101 K)

The Asn101 residue makes part of the dimerization interface in the cap domain, located in helix α4. Asn101 interacts with Lys115 from the other chain by a hydrogen bond, therefore its replacement for a lysine could cause an electrostatic clash with Lys115, disturbing dimerization stability [12]. This rare p.Asn101Lys variant was only reported in a German patient [46].

### Leu104 Val (L104 V)

The Leu104 residue is located in the dimerization interface, in the cap domain of the PMM2 structure. By replacing it with a smaller valine, some hydrophobic interactions that maintain the protein’s dimeric structure can be disrupted [12]. The p.Leu104 Val mutation was reported in a compound heterozygous [p.Leu104 Val/IVS1–1G > A (intervening sequence)] patient. In the first months of life, she presented hypertrophic cardiomyopathy and pericardial effusion. Other symptoms such are anaemia, coagulopathy, hypoglycaemia, hypoalbuminemia, and GE reflux were also observed. Residual activity in fibroblasts was low. The clinical severity can be explained by the fact that the Leu104 residue is highly conserved, and also by the reduced mRNA levels induced by the IVS1–1G > A variant at the splicing site [78].

In a different study, Masunaga et al. [45] identified a Japanese patient with primary ovarian insufficiency (POI) associated with PMM2-CDG showing the p.Leu104 Val/p.Asp12His (c.34G > C) genotype. Moreover, she demonstrated hypotonia, FTT, hypertelorism, strabismus, kyphoscoliosis and endocrine involvement (hypergonadotropic hypogonadism, mild hyperprolactinemia, and hyperthyroglobulinemia).

### Ala108 Val (A108 V)

The Ala108 residue is positioned in helix α4 of the cap domain, more precisely in the dimerization interface. The replacement of this residue for a bulkier valine could result in steric hindrance in this region, and steric clashes with Ser105 located in helix α4 of the other chain of the PMM2 structure, destabilizing the quaternary structure [66].

This missense variant was reported in compound heterozygosity p.Ala108 Val/c.511 dupA in a mildly affected Italian girl with moderate cerebellar hypoplasia [7]. In the same study, the same variant was also found in compound heterozygosity (p.Ala108 Val/70 kb-deletion) in a French patient with mild clinical severity. Biochemical analysis revealed increased serum transaminases without coagulation abnormalities. This patient presented esotropia, hypotonia, hyperlaxity, inverted nipples, feeding problems and cerebellar vermis hypoplasia but no facial dysmorphism.

### Pro113Leu (P113L)

The Pro113 residue resides in the loop that connects the helix α4 with the sheet β6 in the cap domain that constitutes the dimerization interface of the PMM2 structure. The replacement of this residue for a leucine can destabilize this loop, and, consequently, impact protein dimerization [12]. The p.Pro113Leu mutation was reported in compound heterozygosity with the p.Arg141His variant in an individual with severe hyperinsulinemic hypoglycaemia combined with typical clinical manifestations of PMM2-CDG, such as disturbed development, retracted nipples, strabismus, brain hypoplasia, blood coagulation abnormalities, and low levels of thyroid hormones (thyroxine and thyroxine-binding globulin) [13]. Another patient carrying the same allelic combination showed ataxia, brain atrophy, hyporeflexia, disturbed psychomotor abilities, retinitis pigmentosa, and strabismus [10]. In other study, authors [31] reported a compound heterozygous (p.Pro113Leu/p.Phe157Ser) patient with a moderate phenotype and four compound heterozygous patients were associated with a severe clinical phenotype (two carrying p.Pro113Leu/p.Thr237Met, one carrying p.Pro113Leu/p.Arg141His and another carrier of p.Pro113Leu/p.Arg123Gln).

### Phe119Leu (F119L)

The Phe119 residue locates in the dimerization interface, in the cap domain, and binds the Cl^−^ located in the middle of the subunit that may promote dimerization stability in this protein structure. The replacement of the Phe119 impairs this interaction by disrupting some hydrophobic interactions [12, 66]. This missense mutation is more frequent in compound heterozygosity with p.Arg141His and is predominantly prevalent in Scandinavia [4, 36, 37]. By analysing the clinical data of 25 Danish patients, Kjaergaard et al. [37], described that most patients were unable to walk without support, and had serious hypotonia, FTT, feeding difficulties, and liver dysfunction. They also presented impaired development, inverted nipples, subcutaneous fat pads and pericardial effusion in the postnatal period. Some patients presented progressive brain atrophy. In another study, Kjaergaard et al. [36] reported 25 Danish patients with the same genotype (p. Phe119Leu/p.Arg141His). All showed postnatal growth failure. Two had a fatal outcome during the neonatal period and four died during childhood.

### Ile120 Thr (I120 T)

Ile120 is positioned in the dimerization interface in the cap domain, belonging to sheet β6 [12]. Segovia-Falquina et al. [66] reported that the replacement of Ile120 for a threonine could severely impact the dimerization interface, which can explain the impossibility of obtaining a PMM2 protein with this mutation in vitro. Vals et al. [72] reported a patient with a mild phenotype associated with the p.Ile120 Thr/p.Gly228 Cys genotype, describing ataxia, blood coagulation abnormalities, cerebellar vermis hypoplasia, disturbed motor development, impaired speech, FTT, and strabismus. Moreover, Dinopoulos et al. [24] reported a patient with p.Ile120 Thr/p.Val231Met and blood coagulation abnormalities, hemiparesis, and seizures. As previously mentioned, PMM2-CDG patients are typically compound heterozygous, carrying one severe variant that eliminates protein activity and a milder variant [71]. The p.Ile120 Thr variant could be classified as severe because p.Gly228 Cys and p.Val231Met were identified in compound heterozygosity with p.Arg141His known to completely abolish protein activity [7, 66, 68].

### Arg123Gln (R123Q)

Arg123 is located in the cap domain and its side-chain makes two hydrogen bonds with the activator (G16BP). The replacement to a glutamine residue would result in the loss of these interactions, impairing the binding of G16BP to PMM2 [12]. In accordance, Yuste-Checa et al. [82] stated that the p.Arg123Gln pathogenic variant almost abolishes PMM2 catalytic activity. This variant was identified in compound heterozygosity with p.Cys241Ser and a mild clinical phenotype [14, 79]. One patient presented clinical manifestations commonly involved in PMM2-CDG such as impaired development and psychomotor abilities, hypotonia, mild brain atrophy, cerebellar vermis hypoplasia, tremors and mild lipodystrophy. Routine biochemistry was normal, and there was a high PMM2 residual activity in fibroblasts and leukocytes [14]. The second patient showed ataxia, esotropia, hypotonia, impaired mental development, feeding problems due to GE reflux and retracted nipples [79].

### Val129Leu (V129L)

Val129 residue is located in a hydrophobic cluster in sheet β7 in the cap domain. Its substitution for a bulkier leucine could destabilize the proper folding of the cap domain region [12]. Wurm et al. [81] reported two compound heterozygous patients (p.Val129Leu/161_162 insG) with a severe phenotype. Both patients showed hydrops fetalis and oedema during foetal development, and, after birth, hydrops n with severe amegakaryocytic thrombocytopenia and hypoglycaemia. In addition, both had a rapid progression of hypertrophic cardiomyopathy, resulting in an acute onset of cardiological failure with death after a few weeks. Although p.Val129Leu can have a role in diminishing the PMM2 activity, it is also likely that the poor outcome resulted from the null 161_162 insG mutation that completely abolishes protein activity.

### Val129Met (V129M)

According to Segovia-Falquina et al. [66], the replacement of Val129 for a bulkier methionine can result in steric hindrance since the side chain of valine is projected towards the nonpolar inner core of the cap domain. Additionally, it could disturb the hydrogen bound between the side-chain of the near Asn128 and the activator (G6BP), compromising substrate binding. In line with these observations, the authors observed that this mutation reduces by more than half the PMM2 activity levels. The p.Val129Met variant is commonly associated with compound heterozygosity with p.Arg141His mutation and a severe phenotype [7, 8]. As reported by Barone et al. [8], this patient showed common PMM2-CDG symptoms including severe neurological and multisystem involvement such as ataxia, brain atrophy, osteopenia, hepatic steatosis, and abnormal levels of blood coagulation factors. Furthermore, Barone et al. [7] described five Italian patients with the same genotype that were also characterized by a severe phenotype, and two compound heterozygous (p.Leu32 Arg/p.Val129Met) Italian patients with mild clinical severity. Finally, Perez-Duenas et al. [53] reported one compound heterozygous (p.Val129Met/IVS3 + 2 C > T) patient with a severe phenotype resulting in early fatal outcome.

### Pro131 Ala (P131 A)

The Pro131 residue is located in the turn between sheet β7 and α5 helix. The replacement for a more permissive alanine residue can result in the misfolding of the cap domain region [12]. This rare mutation was only reported by Le Bizec et al. [42] in a compound heterozygous (p.Pro131 Ala/p.Arg141His) French patient but no description of the phenotype is available.

### Ile132Phe (I132 F)

Ile132 residue is situated in the cap domain and the replacement for a bulkier phenylalanine can disrupt some hydrophobic interactions, impacting the proper folding of the cap domain in protein structure [12]. Previous data revealed that this mutation does not have a significant impact on protein activity but confers less thermostability to PMM2 [42]. The same study reported a French patient with p.Phe113Leu on one allele and p.Glu197 Ala (c.590 A > C)/p.Ile132Phe on the other one.

### Ile132 Thr (I132 T)

Ile132 residue is situated in the cap domain in the PMM2 structure, and substitution for a polar threonine can disturb the proper folding of this region [12]. Gonzalez-Dominguez et al. [30] identified a Mexican patient with this variant in combination with the p.Arg141His variant. The clinical symptoms of the patient were hypotonia, retarded development and psychomotor abilities, feeding problems, and recurrent seizures. Strabismus, facial dysmorphism, fat pads, and hyperprolactinemia were also observed. Furthermore, de Lonlay et al. [23] reported two patients, one carrying the p.Ile132 Thr/p.Arg123Gln variant and the other the p.Arg141His/p.Ile132 Thr genotypes. Both showed FTT and similar neurological manifestations, including brain hypoplasia, disturbed psychomotor abilities, areflexia, hypotonia, and strabismus as well as other organ involvement (hepatomegaly, tubulopathy). Only the first patient showed dysmorphism and pericardial inflammation. Finally, Shanti et al. [67] identified a patient with the p.Ile132 Thr/p.Phe207Ser genotype and feeding problems due to GE reflux as well as hypotonia, hepatomegaly, and hypothyroidism. No fat pads were observed and neuroimaging was normal.

### Glu139Lys (E139 K)

The Glu139 residue is found in the helix α5 of the cap domain. Its replacement by a positively charged lysine can lead to the destabilization of this helix due to the different charge properties of these residues [12]. The p.Glu139Lys variant shows a high residual PMM activity and less thermolability compared to the WT protein [42]. It was first reported in compound heterozygosity with p.Arg141His in two French patients [76]. The same genotype was also reported in two other patients. Both exhibited neurological symptoms such as areflexia, hypoplasia, hypotonia, disabled psychomotor capacities and seizures [23]. Romano et al. [65] described three patients with PMM2-CDG and conotruncal heart defects. One patient with p.Glu139Lys/p.Arg141His was prenatally diagnosed with Tetralogy of Fallot. This patient experienced feeding problems during infancy, requiring percutaneous endoscopic gastrostomy, hypotonia, inverted nipples and cerebellar atrophy/hypoplasia.

### Arg141His (R141H)

The Arg141 residue, in the PMM2 structure, is located in helix α5 on the cap domain where it binds the substrate (G16BP) by a charged interaction with the 1-phosphate. Replacing this residue with a histidine would severely impact PMM2 activity since it would disrupt this interaction, impairing substrate binding [12]. In accordance, in vitro analysis evaluated that the p.Arg141His mutation results in null activity [39, 42, 77, 82].

Barone et al. [7] reported 14 Italian patients with a severe phenotype, who were compound heterozygous for p.Arg141His. All cases required aid to walk and had cerebellar hypoplasia and eye disorders. In addition, two had epilepsy, six suffered from stroke-like episodes, and ten manifested microcephaly.

de Lonlay et al. [23] reported 17 compound heterozygotes for p.Arg141His, which is common in patients with French and Portuguese ancestry. Eight of these patients manifested a predominant neurological clinical spectrum, and nine presented a multisystem involvement. The first group described early manifestations of cerebellar hypoplasia, psychomotor problems, strabismus and manifestations in the second year of life, such as neuropathy and retinitis pigmentosa. The second group of patients manifested an early onset of neuronal and extraneuronal disabilities, most with fatal outcomes. Strabismus and cerebellar hypoplasia were uncommon, while on the other hand, hepatic and renal abnormalities were frequent. Both groups of patients presented dysmorphic features including fat pads and inverted nipples.

Stefanits et al. [68] reported a compound heterozygous (p.Arg141His/p.Gly228 Cys) patient with a brain haemorrhage and a malignant brain tumour in addition to the typical PMM2-CDG phenotype.

Finally, Bastaki et al. [9] reported a compound heterozygous p.Arg141His/p.Val43Met (c.127G > A) patient with some dysmorphism, esotropia, hypermetropia, hypotonia, disabled development and increased serum transaminases.

### Phe144Leu (F144L)

The Phe144 residue is located in the helix α5 in the cap domain. The phenylalanine substitution for a leucine, which is a smaller amino acid, in a region occupied mostly by hydrophobic side-chains and π-π interactions, can affect the proper folding of the cap domain [12].

Kondo et al. [40] reported two compound heterozygous (p.Arg238Pro/p.Phe144Leu patients. One of them had a rapid onset after birth, with muscular hypotonia and impaired motor development. Later, she also suffered from recurrent stroke-like episodes. At the age of eight years, she manifested muscle hypotonia, tremors, ataxia, low height, dysmorphic facial traits, strabismus, and could only walk with support. The other patient presented feeding difficulties, severe delayed development, muscular hypotonia, and mild brain atrophy. She also had episodes of seizures and hemiparesis due to several head injuries. At 18 years of age, she was not capable of speaking and suffered from hypergonadotropic hypogonadism.

In the same study, Kondo et al. [40] also described two compound heterozygous [p.Tyr229Ser (c.677 C > A)/p.Phe144Leu] Japanese patients. One patient showed ataxia, hypotonia, disabled mental development, recurrent stroke-like episodes, brain atrophy and increased serum transaminases. The other patient manifested hypotonia and decreased motor abilities in the first year of life. Both were able to walk unaided in adolescence and required special education.

### Asp148 Asn (D148 N)

The Asp148 residue is located in helix α5, in the cap domain. The replacement by a positively charged arginine could disrupt the folding of helix α6 since it would remove the negative charge that serves to cap this helix at its N-terminus [12]. The p.Asp148 Asn variant was reported in a compound heterozygous (p.Phe183Ser/p.Asp148 Asn) female with disabled development, fat pads, feeding difficulties, hypotonia, dysmorphism, strabismus, and spine abnormalities [33]. Additionally, a compound heterozygous (p.Cys9 Tyr/p.Asp148 Asn) characterized by a mild phenotype was mentioned above [79].

### Ile153 Thr (I153 T)

Ile153 is located in the cap domain region and its substitution for a polar threonine could disrupt the proper folding of the cap domain [12]. Since this residue is located at the hydrophobic inner core of the cap domain, and results in an unstable protein with almost no protein activity, it is classified as a severe variant [66]. It was identified in compound heterozygosity with p.Arg141His [23]. One of the patients demonstrated neurological involvement such as brain hypoplasia, hypotonia, strabismus, disturbed psychomotor abilities and seizures, as well as some multisystem involvement such as diarrhoea, pericarditis and renal cysts [23]. Furthermore, [31] reported one compound heterozygous patient (p.Arg123Gln/p.Ile153 Thr) that revealed a severe phenotype with multisystem involvement with recurrent infections, abnormal development and growth, retinitis pigmentosa and peripheral neuropathy.

### Phe157Ser (F157S)

The Phe157 residue is located in helix α6, more precisely, in the cap domain. The replacement by a serine can disrupt the proper folding of the PMM2 structure in this region [12]. Vega et al. [73] concluded that the p.Phe157Ser missense variant nullifies the enzymatic activity of PMM2.

This variant accounts for different phenotypes and different clinical severities. On one hand, it was described in two compound heterozygotes (Phe157Ser/p.Cys241Ser) with a mild phenotype [31, 60]. On the other hand, a severe phenotype with early death was reported in two compound heterozygotes [p.Phe157Ser/p.Asp65 Tyr [11], p.Phe157Ser/p.Val231Met [60]].

### Arg162 Trp (R162 W)

Arg162 is a charged residue that is situated in the cap domain. The replacement by a hydrophobic tryptophane residue would possibly induce a negative impact on protein conformation. This could occur through the introduction of a bulkier and nonpolar side chain, which could cause a shielding effect [12]. The p.Arg162 Trp missense variant was detected in two patients presenting compound heterozygosity with p.Arg123X (367 C > T) and p.Arg141His variants, characterized by a moderate phenotype. This can be explained by the fact that p.Arg162 Trp does not completely abolish protein activity [60]. In fact, Yuste-Checa et al. [82] reported that the p.Arg162 Trp mutation has a mild impact on the PMM2 protein since it retains almost half of the activity of the WT protein. In accordance, Grünewald et al. [31] had previously identified a compound heterozygous (p.Arg141His/p.Arg162 Trp) patient with the same clinical severity.

### Phe183Ser (F183S)

As Segovia-Falquina et al. [66] described, the hydrophobic Phe183 residue is located in the cap domain of the PMM2 structure, and its side chain would interact with the core domain following the closure of the subunit. This would result in low residual protein activity when the phenylalanine is replaced by a serine at the amino acid 183. On the other hand, Briso-Montiano et al. [12] predicted that this mutation could modify the function of linker- 2, which is important for the catalytical process of PMM2. It was reported in one homozygous (p.Phe183Ser/p.Phe183Ser) and in seven compound heterozygous (p.Phe183Ser/p.Arg141His) patients showing neurological involvement, mild peripheral neuropathy and mild liver dysfunction [25].

### Gly186 Arg (G186R)

Gly186 residue belongs to the linker- 2 (aa 185–188) substructure in the PMM2 structure. The substitution for a less flexible arginine residue would increase the rigidness of the linker, which could impair the catalytical process of the PMM2 structure [12]. This missense variant was reported in homozygosity (p.Gly186 Arg/p.Gly186 Arg) in two individuals from a consanguineous Chinese family with premature ovarian insufficiency. Curiously, none of these patients presented the most common traits of PMM2-CDG. By utilizing in silico strategies, it was determined that this variant was located in a highly conserved protein site and characterized as potentially pathogenic. Moreover, the authors showed that it reduces PMM2 activity [52].

### Asp188Gly (D188G)

Asp188 residue constitutes the linker- 2 of the PMM2 structure. The replacement for a glycine residue would increase the flexibility of linker- 2, which can negatively affect the catalytic process. It was identified in a compound heterozygous (p.Asp188Gly/p.Leu35X) patient with FTT, fat pads, inverted nipples, strabismus, ascites, pericardial effusion, hepatic involvement, hypoalbuminemia, thrombocytopenia, and renal involvement, with early fatal outcome [70]. Pirard et al. [55] also reported four compound heterozygous (p.Asp188Gly/p.Arg141His) patients with a severe phenotype and early death.

### Phe207Ser (F207S)

The nonpolar Phe207 residue is located in sheet β10, in the core domain. Its replacement by a polar serine could affect the hydrophobic clusters of side chains and π-π interactions in that region, which could lead to unproper folding of the core domain. Previous data demonstrated that the p.Phe207Ser variant results in a protein with null activity [73]. It was reported in a compound heterozygous [p.Tyr76 Cys (c.227 A > G)/p.Phe207Ser] patient with hepatopathy in the neonatal period, dysmorphism, cerebellar hypoplasia, severe encephalopathy and a fatal outcome in early adolescence. Another compound heterozygous (p.Phe207Ser/p.Asn216Ile) patient manifested retracted nipples, convulsions, disabled psychomotor development, obesity and cerebellar hypoplasia [11].

### Gly208 Ala (G208 A)

Gly208 residue is situated in the core domain, on the PMM2 structure, and its replacement for an alanine residue could displace the near Asp209 residue, which coordinates the catalytical Mg^2+^, and, consequently, impacting the catalytic process [12]. This variant was found in a male patient in compound heterozygosity with the p.Arg141His replacement. The patient presented anaemia, coagulation abnormalities, ventricular septum defect and pericardial effusion, hepatic and renal involvement. Seizures and multiorgan failure resulted in death at eight months of age [2]. Another patient with the same genotype showed multiorgan involvement and a lethal outcome due to severe pericardial effusion and left ventricle failure [32, 33].

### Asn216Ile (N216I)

The Asn216 residue belongs to the β10‐α8 loop (aa 212–217) that coordinates the catalytical Mg^2+^. Its substitution for an isoleucine could displace the Asp209, one of the residues that form the coordination complex with the catalytical Mg^2+^, which results in binding inhibition of this cofactor, thus impacting the catalytic activity of PMM2 [12]. This variant was reported in compound heterozygosity with p.Arg141His and associated with a severe phenotype [7]. It was also described in a homozygous patient with a mild phenotype (p.Asn216Ile/p.Asn216Ile) [49]. The patient showed strabismus, macrosomia, mild psychomotor disability, hypotonia, recurrent respiratory tract infections including bronchopneumonia, brain hypoplasia and dilatation of the 4 th ventricle. Fat pads, retracted nipples, pericardial effusion, and cardiomyopathy, typical PMM2-CDG symptoms were not reported.

### Asp223Glu (D223E)

Asp223 is one of the residues that is located in the α8-β11 loop (aa 223–226), with one of the two Mg^2+^ atoms on the PMM2 structure. Replacing this residue with a glutamate could affect the interactions with the Mg^2+^, which can impact the correct folding of the protein [12]. As reported by Kjaergaard et al. [38], this variant was identified in a Danish patient leading to the nearly total loss of PMM activity in fibroblasts, but the maternally inherited variant could not be found.

### Asp223 Asn (D223 N)

Similarly to the previously mentioned p.Asp223Glu variant, the p.Asp223 Asn variant could impair the coordination of the structural Mg^2+^, resulting in unproper folding of PMM2 [12]. It was reported in three compound heterozygous Italian patients [two carrying p.Gly15Glu (c.44G > A)/p.Asp223 Asn and one carrying p.Asp223 Asn/p.Arg141His], with a severe phenotype. The first two patients presented intellectual disability, inability to walk without support, stroke-like episodes, visual defects, severe microcephaly and brain hypoplasia. The other patient had severely impaired intellectual development and microcephaly [7].

### Thr226Ser (T226S)

The Thr226 residue coordinates the Mg^2+^ with a structural function. Therefore, replacing it with a serine could disturb some hydrophobic interactions and the interaction with the Mg^2+^ [66]. This could reduce the stability of this complex and negatively affect the proper folding of the protein [12].

The replacement of threonine at this amino acid residue was reported in a compound heterozygous (p.Thr226Ser/p.Arg141His) patient exhibiting a moderate phenotype [60]. Grünewald et al. [31] reported a patient with the same genotype and a mild phenotype: hypotonia, strabismus, psychomotor disability and cerebellar hypoplasia.

### Gly228 Cys (G228 C)

In the PMM2 structure, the Gly228 residue is situated in sheet β11, in the core domain where it makes a hydrogen bond with Thr226, which constitutes the metal complex with the structural Mg^2+^ with Phe221 and Asp223. Disturbing this coordination complex could cause abnormal folding of the protein structure [12]. The p.Gly228 Cys variant was reported in a compound heterozygous (p.Ile120 Thr/p.Gly228 Cys) patient with a mild phenotype [72], and another compound heterozygous individual (p.Arg141His/p.Gly228 Cys) showed brain haemorrhage [68], as reported above.

### Val231Met (V231M)

By analysing the PMM2 experimental structure, Segovia-Falquina et al. [66] predicted that the replacement of Val231 residue for a bulkier methionine would provoke steric hindrance since Val231 makes hydrophobic interactions in the inner core domain. Since the p.Val231Met (c.691G > A) variant is often found in compound heterozygosity with the severe variant p.Arg141His, it can be assumed as mild [6, 7, 23, 66]. However, Barone et al. [7] reported three patients with these variants and severe intellectual disability, incapability of walking without support, microcephaly and severe cerebellar hypoplasia. de Lonlay et al. [23] also identified three patients with the same missense variants. They showed a multisystem involvement: neurological manifestations as seen in PMM2-CDG, vomiting, and renal problems. Two of these patients had cardiological involvement and an early fatal outcome. Another two patients with the same genotype were identified by Asteggiano et al. [6], and suffered from multiorgan involvement leading to early death. Aronica et al. [5] reported a compound heterozygous (p.Val231Met/Thr237 Arg) patient that presented a severe phenotype in the neonatal period and a typical PMM2-CDG phenotype. Cardiological and respiratory failure led to a lethal outcome at one month of age. Contrastingly, Vals et al. [72] reported three compound heterozygous (p.Val231Met/p.Arg239 Trp) patients with a mild phenotype and an appropriate cognitive development.

### Thr237 Arg (T237R)

Thr237 resides in helix α9, in the core domain and makes a hydrogen bond with Thr16 that belongs to the β1-α1 loop that coordinates the catalytical Mg^2+^. The replacement of Thr237 for a bulkier arginine would probably cause steric hindrance, leading to disturbed folding of the core domain [12]. Moreover, Kjaergaard et al. [39] reported that the p.Thr237 Arg (c.710 C > G) variant would completely abolish the PMM2 enzymatic activity.

A severe phenotype was reported in a patient with p.Thr237Met (c.710 C > T) on one allele and p.Thr237 Arg/p.Ala233 Thr (c.697G > A) on the other, resulting in almost total absence of PMM activity in fibroblasts [31]. In contrast, a mild phenotype with mild intellectual impairment and moderate cerebellar hypoplasia was described in a compound heterozygous (p.Thr237 Arg/p.Cys241Ser) patient [7].

### Thr237Met (T237M)

The replacement of Thr237 for a methionine could disrupt the hydrogen bond between Thr237 and Thr16, which could impair catalysis, besides disturbing protein conformation or folding stability [12]. Yuste-Checa et al. [82] stated that PMM2 with the p.Thr237Met variant would result in a protein with a high ability of dimerization and intermediate catalytical activity.

As to the clinical impact, Imtiaz et al. [33] reported a compound heterozygous (p.Arg141His/p.Thr237Met) patient, with ataxia, severe muscle hypotonia, mild dysmorphism, impaired development, FTT, tremors, and mild retinal dysfunction. Tayebi et al. [69] identified a compound heterozygous patient with p.Thr237Met and the deletion-insertion c.565–571 delAGAGAT insGTGGATTTCC. Its phenotype comprised amblyopia, abnormal fat distribution, FTT during infancy, dysmorphic traits, strabismus, retracted nipples, impaired development, GE reflux, muscle hypotonia, inability to walk without support and mild brain hypoplasia. PMM residual activity was slightly decreased.

### Arg238Pro (R238P)

Arg238 resides in helix α9, in the core domain, and its substitution for a proline could provoke misfolding of the protein structure since it would disarrange some hydrophilic interactions that maintain structure stability [12]. The p.Arg238Pro missense variant was mentioned above in four patients in association with p.Phe144Leu [40]. This variant was also reported by Choi et al. [17] in a compound heterozygous [p.Arg194X (c.580 C > T)/p.Arg238Pro] Korean patient with FTT, recurrent non-epileptic seizures, impaired development and hepatomegaly. Serum transaminases were increased and serum ceruloplasmin was low.

### Arg239 Trp (R239 W)

The Arg239 residue constitutes helix α9 in the core domain of the PMM2 structure and makes a hydrogen bond with Asp236, which in turn links by another hydrogen bond with Thr232 that is positioned before helix α9. Therefore, the replacement of Arg239 by a tryptophan could result in the loss of these polar contacts, and, consequently, in the disruption of helix α9. Another possibility would be the resultant shielding effect by the larger side chain of tryptophan that could be projected towards the solvent [12]. This variant was reported in five compound heterozygous (p.Val231Met/p.Arg239 Trp) patients, three with a mild [72], and two with a mild/moderate phenotype [31].

### Cys241Ser (C241S)

The Cys241 residue is located in the core domain and its side chain is directed towards the hydrophobic inner face of the core domain [12]. Its replacement by polar serine could disrupt some hydrophobic interactions in the core domain region. According to Vega et al. [73], the p.Cys241Ser mutation presents more than half of the WT activity and could be considered a mild mutation. In accordance, Vals et al. [72] identified two compound heterozygous (p.Cys241Ser/p.Arg141His) patients with only mildly impaired motor development, and without coagulation abnormalities or hepatic dysfunction. Quelhas et al. [60] also reported two patients with the same genotype and a mild phenotype.

## Conclusions

Genotype–phenotype correlations in PMM2-CDG are a difficult challenge due to the relatively high number of variants, and the broad phenotypical spectrum. The dimeric nature of PMM2 and the impact of each combination in the protein half-life also contribute to an increased difficulty in establishing these correlations. Moreover, the critical role of PMM2 in the sugar doner synthesis and its impact on a myriad of metabolic interactions expands the clinical effect of *PMM2* genotype [58]

Structural analyses could be a valuable approach to investigating disease-associated variants'molecular basis. By analysing the mechanisms of pathogenicity, that impact PMM2 structure and function, novel insights into PMM2-CDG could be obtained. At other level, online prediction tools like PolyPhen- 2 (Polymorphism Phenotyping v2) [1], PROVEAN (Protein Variation Effect Analyzer) (ref) [18] and SIFT (Sorting Intolerant From Tolerant) [50] that estimate the impact of missense mutations on the proteins function and structure can also offer valuable theoretical insight [15, 20, 51]. In addition, CADD (Combined Annotation Dependent Depletion) [35, 63] can complement these tools by providing a genome-wide scoring system that integrates multiple annotations to assess the deleteriousness of variants, including single nucleotide variants, indels, and structural variants, enhancing variant interpretation. Prior research used a systems biology approach, using transcriptomic data to create a computational model for PMM2-CDG. Moreover, recent studies found evidences that the root causes of PMM2-CDG may be similar in various cell types and could overlap with other CDGs [28]. The theory suggests that problems in glycosylation processes could affect secondary cellular pathways similarly, regardless of the genetic issue. This presents a chance for treatments that target shared pathways among different CDGs, potentially inspiring more researchers to explore this promising avenue and helping more patients access new therapies.

## Data Availability

Not applicable.
